# Semantic characteristic grading of pulmonary nodules based on deep neural networks

**DOI:** 10.1186/s12880-023-01112-4

**Published:** 2023-10-13

**Authors:** Caixia Liu, Ruibin Zhao, Mingyong Pang

**Affiliations:** 1 College of Intelligent Education, Jiangsu Normal University, Xuzhou, Jiangsu China; 2 Institute of EduInfo Science and Engineering, Nanjing Normal University, Nanjing, China

**Keywords:** Semantic characteristic, Pulmonary nodule, Malignancy, Correlation, Deep neural network

## Abstract

**Background:**

Accurate grading of semantic characteristics is helpful for radiologists to determine the probabilities of the likelihood of malignancy of a pulmonary nodule. Nevertheless, because of the complex and varied properties of pulmonary nodules, assessing semantic characteristics (SC) is a difficult task.

**Method:**

In this paper, we first analyze a set of important semantic characteristics of pulmonary nodules and extract the important SCs relating to pulmonary nodule malignancy by Pearson's correlation approach. Then, we propose three automatic SC grading models based on deep belief network (DBN) and a multi-branch convolutional neural network (CNN) classifier, MBCNN. The first DBN model takes grayscale and binary nodule images as the input, and the second DBN model takes grayscale nodule images and 72 features extracted from pulmonary nodules as the input.

**Results:**

Experimental results indicate that our algorithms can achieve satisfying results on semantic characteristic grading. Especially, the MBCNN can obtain higher semantic characteristic grading results with an average accuracy of 89.37%.

**Conclusions:**

Quantitative and automatic grading of semantic characteristics proposed in this paper can assist radiologists effectively assess the likelihood of pulmonary nodules being malignant and further promote the early expectant treatment of malignant nodules.

## Introduction

Lung cancer is one of the most common cancers in the society of human beings, and it is also the leading cause of cancer-related deaths in the world [1]. According to an analysis report about the prevalence of malignant tumors published by the National Cancer Center of China in 2019, lung cancer ranks first in the incidence of malignant tumors in the country [2]. A pulmonary nodule is an early manifestation of lung cancer. Thus, early diagnosis and treatment of pulmonary nodules can effectively improve the survival rate of patients suffering from lung cancers. Computed tomography (CT) is a widely used imaging technique for the detection of pulmonary nodules. Clinical experiments illustrate that lung cancer can be diagnosed one year earlier by CT rather than a chest X-ray. A patient can obtain more survival time of 0.019 years and reduce the mortality rate by 15% with each scan received. Semantic characteristics (SCs) of pulmonary nodules, e.g. spiculation, lobulation, margin, etc., are important features for assisting radiologists in determining the malignancy of pulmonary nodules in CT images [3, 4]. However, current computer-aided diagnosis systems (CAD) rarely offer SCs and their corresponding ratings [5]. SC grading mainly depends on the naked-eye observation of radiologists. It is also a time-consuming and boring process.

Lung Image Database Consortium (LIDC) provides nine SCs of pulmonary nodules including subtlety, internal structure, calcification, sphericity, margin, lobulation, spiculation, texture, and malignancy [6]. These SC ratings are based on radiologists' experience [7]. Quantitative and automatic grading of SCs is greatly helpful for radiologists to interpret pulmonary nodules and predict the likelihood of nodule malignancy with CAD. In this paper, we analyze the relationships between a set of SCs and the malignancy of pulmonary nodules and then introduce three methods for SC grading based on neural networks. In addition, we discuss the factors that influence the accuracy of SC grading. The main contributions of our work in this paper are:

- We analyze the correlations of SCs with the malignancy of pulmonary nodules, which is helpful to the nodule classification and malignancy estimation.
- We propose neural network-based automatic SC grading models, which are conducive to assisting radiologists in interpreting pulmonary nodule images and making decisions.

## Related work

The SCs of pulmonary nodules in CT, such as lobulation and spiculation, are of great value in the differentiation and diagnosis of benign and malignant nodules. Choi et al. [8] argued that malignant nodules were more likely to have irregular, lobulated, or spiculated margins due to the spread of malignant cells within the pulmonary interstitium. Liang et al. [9] found that lobulation is an important imaging feature in differentiating the pathologic properties of solitary pulmonary nodules (SPN) lobulation on the edge of SPN can strongly suggest malignant lesions. On the contrary, the possibility of malignant lesions without lobulation on the edge of SPN is greatly reduced. Ye et al. [10] explored the value of multiple signs such as internal features (vacuolar sign, calcification) and marginal features (lobulation and spiculation) in the differential diagnosis of benign and malignant SPN. They found that the lobulation and spiculation had high sensitivity, specificity, and accuracy in the differential diagnosis of benign and malignant SPN.

In recent years, many algorithms have been proposed for detecting and grading of SCs. Spiculation is one of the most important characteristics to assist radiologists in estimating pulmonary nodule malignancy. Choi et al. [8] presented an interpretable and parameter-free technique to quantify the spiculation using an area distortion metric obtained by conformal (angle-preserving) spherical parameterization. Tao et al. [11] proposed a quantitative evaluation method of lung nodule spiculation combining super-resolution reconstruction-based image enhancement techniques with lung nodule segmentation algorithms. Zhang et al. [12] put forward a pulmonary nodule spiculation recognition algorithm. In the model, morphological component analysis was employed to segment pulmonary nodules, and a semisupervised generative adversarial network was built to recognize pulmonary nodule spiculation. Xin et al. [13] developed a nodule subtlety degree assessment technique, where subtlety was defined in terms of 'difficulty of reader detection'. They extracted a total of 30 features from each of the 3D nodules. The subtlety degree was assessed by an artificial neural network classifier with features extracted from 407 nodules. Perconti et al. [14] quantified lesion subtlety with a salience and conspicuity measure that was correlated to observer perception. Lobulation is another important CT imaging sign and is dependent on the ingrowth of connective tissue septa containing fibroblasts derived from perithymic mesenchyme [15]. Lobulation is normally related to malignant lesions, though it also occurs in up to 25% of benign nodules [16, 17]. Choi et al. [18] presented an end-to-end deep learning model based on multi-class Voxel2Mesh extension to segment nodules, classify sharp/spiculation and curved/lobulation, and perform malignancy prediction finally. Texture analysis of lung computed tomography (CT) images is a critical tool for some lung diseases. Yang et al. [19] explored characterizing lung textures with sparse decomposition from texton dictionaries, using different regularization strategies and extending the sparsity-inducing constraint to the construction of the dictionaries. Vishraj et al. [20] presented shape-based features to quantify the amount of fibrotic and nodular components in a lung tissue pattern utilizing random forest and logistic regression. Varutbangkul et al. [21] proposed a grading method of SCs on spiculation, lobulation, margin, and sphericity. Radiologic visualization of calcification within lung cancer is uncommon and may cause confusion and misdiagnosis. The presence of calcium within pulmonary lesions on radiologic examinations is an important factor in assessing the malignancy of lesions [22]. For example, calcification inside lung nodular shadows is considered a negative finding for malignant tumors [23]. However, there is little research on calcification grading, as well as margin grading.

The performance of the CADx system depends on the accurate estimation of lesion features. Ratings of SCs are important ways of estimating pulmonary nodule malignancy. Although many researchers have argued that the SCs influence the probability of malignancy, the research focused on individual characteristics. In addition to the common semantic characteristics, such as spiculation and lobulation, whether other semantic characteristics are associated with malignant nodules or not, lacks a more complete investigation. In addition, there is little research about the grading of calcification, sphericity, margin, and so on. Our research aims to find the relationships between SCs and pulmonary nodules' malignancy and exploit an automatic SC grading method with machine learning technology according to radiologists' annotations available.

## Materials and Methods

We first introduce the dataset used in this study, and then we extract the important SCs that influence the diagnosis of lung cancers by calculating the correlations between them and the malignancy of pulmonary nodules. Finally, we construct three neural network-based models for SC grading.

### Dataset

Lung Image Database Consortium image collection (LIDC-IDRI) [24] is initiated by the National Cancer Institute (NCI), further advanced by the Foundation for the National Institutes of Health (FNIH), and accompanied by the Food and Drug Administration (FDA) through active participation. The database provides an authoritative and open standard for pulmonary cancer research [25–27]. It consists of diagnostic and lung cancer screening thoracic computed tomography (CT) scans with marked-up annotated lesions. It contains 1018 cases, and four experienced thoracic radiologists mark the images in each case. Each suspicious lesion is categorized as a non-nodule, a nodule < 3 mm, or a nodule $\ge$ 3 mm in diameter in the long axis of nodules. LIDC-IDRI provides the boundary information of nodules with diameters from 3 mm to 3 cm, and up to 4 radiologists give the ratings of nine SCs.

### Malignancy-related SC extraction

In this section, we conduct experiments to find the relationship between SCs and malignant nodules, and further extract the malignancy-related SCs. The process includes two steps: sample selection, and the calculation of correlation between SCs and nodule malignancy.

#### Sample selection

We randomly select a group of nodules with a diameter ranging from 3 mm to 3 cm from LIDC-IDRI. Nine SCs including subtlety (C1), internal structure (C2), calcification (C3), sphericity (C4), margin (C5), lobulation (C6), spiculation (C7), texture (C8), and malignancy (C9) of these nodules are annotated by up to four radiologists (N1…N4) as listed in Table 1 [28]. By analyzing the SCs of nodules, we find that the SC ratings of the first and second radiologists are relatively similar and stable. So we adopt a strategy of taking the averaged value of ratings of the first two radiologists as the leading grading judgment, supplemented by those of other radiologists. The nodules without SCs provided by the first radiologist are discarded in the process of nodule sample selection.

**Table 1:** Examples of SC ratings annotated by radiologists

Nodule	N	C1	C2	C3	C4	C5	C6	C7	C8	C9
	**N1**	**5**	**1**	**6**	**3**	**3**	**2**	**2**	**3**	**5**
	**N2**	**5**	**1**	**6**	**4**	**3**	**2**	**2**	**5**	**5**
	N3	3	1	6	2	3	3	2	4	3
	N4	5	1	6	5	4	1	5	4	4
	**N1**	**3**	**1**	**6**	**4**	**5**	**1**	**1**	**5**	**2**
	**N2**	**2**	**1**	**3**	**3**	**5**	**1**	**1**	**5**	**1**
	N3	2	1	3	2	5	1	1	5	1
	N4	5	1	3	5	5	1	1	5	1
	**N1**	**5**	**1**	**6**	**3**	**4**	**2**	**2**	**5**	**4**
	**N2**	**5**	**1**	**4**	**3**	**4**	**1**	**1**	**4**	**3**
	N3	5	1	6	5	5	1	1	5	5
	N4	3	1	6	5	5	1	1	5	2
	**N1**	**3**	**1**	**6**	**4**	**5**	**1**	**1**	**5**	**2**
	**N2**	**3**	**1**	**6**	**5**	**5**	**1**	**1**	**5**	**3**
	N3	5	1	6	5	5	1	1	4	3
	N4	3	1	6	5	5	1	1	5	2

According to the sample selection strategy, 3091 pulmonary nodule images are extracted from 200 cases. The corresponding SC rating information is exhibited in Table 2. For example, subtlety is measured on a scale of 1 to 5 (T = 5), and there are 129 nodule images with a subtlety rating of 1.

**Table 2:** Statistics of SC ratings on sample nodules

Ratings	1	2	3	4	5	6	T
**SC**							
Subtlety	129	174	597	672	1519	-	5
InternalStructure	3075	1	1	14	-	-	4
Calcification	0	10	148	18	46	2869	6
Sphericity	13	218	986	928	946	-	5
Margin	135	241	450	937	1328	-	5
Lobulation	1401	800	423	365	102	-	5
Spiculation	1677	528	406	220	260	-	5
Texture	149	38	129	267	2508		5
Malignancy	257	779	901	579	575	-	5

#### Correlation calculation

We analyze the correlation between the first eight SCs and the malignancy, C9, on 201, 400, 608, and 807 lung nodules with Pearson's correlation coefficient, respectively. The ratings of calcification and internal structure are normalized into five ratings for more effective and uniform processing with the correlation calculation approach.

We randomly select a group of malignant and benign nodules with several corresponding SCs from the dataset. From Table 3, we can find that the malignant nodules usually have larger C1, C3, C6, and C7 and comparatively small C4 and C5. Benign nodules often have smaller C6 and C7, as well as bigger C4 and C5. In order to explore the relationship between SCs and malignancy, we use Pearson's correlation approach to calculate their correlation.

**Table 3:** Nodules with their SCs

Nodule	C1	C3	C4	C5	C6	C7	C9
	5	6	4	3	4	5	5
	5	6	2	3	3	5	5
	5	6	3	3	5	3	5
	3	6	5	5	1	1	2
	5	3	5	5	1	1	1
	5	3	5	5	1	1	1

Assume that there are two sets of characteristic ratings: $X=({x}_{1},{x}_{2},{x}_{3},{x}_{4},{x}_{5},)$ and $Y=({y}_{1},{y}_{2},{y}_{3},{y}_{4},{y}_{5},)$, their Pearson’s correlation coefficient is expressed as

1 $$\rho \left(X,Y\right)=\frac{E(XY)}{{\sigma }_{x}{\sigma }_{y}}=\frac{{\sum }_{i-1}^{n}\left({x}_{i}-\overline{x }\right)\left({y}_{i}-\overline{y }\right)}{\sqrt{{\sum }_{i-1}^{n}{\left({x}_{i}-\overline{x }\right)}^{2}}{\sum }_{i-1}^{n}{\left({y}_{i}-\overline{y }\right)}^{2}}$$

where $E(XY)$ is the cross-correlation between $Y$ and $Y$. ${\sigma }_{x}^{2}=E\left({X}^{2}\right)$, ${\sigma }_{y}^{2}=E\left({Y}^{2}\right)$, $\overline{x }$ and $\overline{y }$ are variances of $X$ and $Y$, mean values of $X$ and $Y$, respectively. $\rho$ indicates the strength of the linear relationship between $X$ and $Y$, and $0\le {\rho }^{2}(X,Y)\le 1$. A bigger $\rho$ often means a stronger correlation between $X$ and $Y$.

**Fig. 1:**
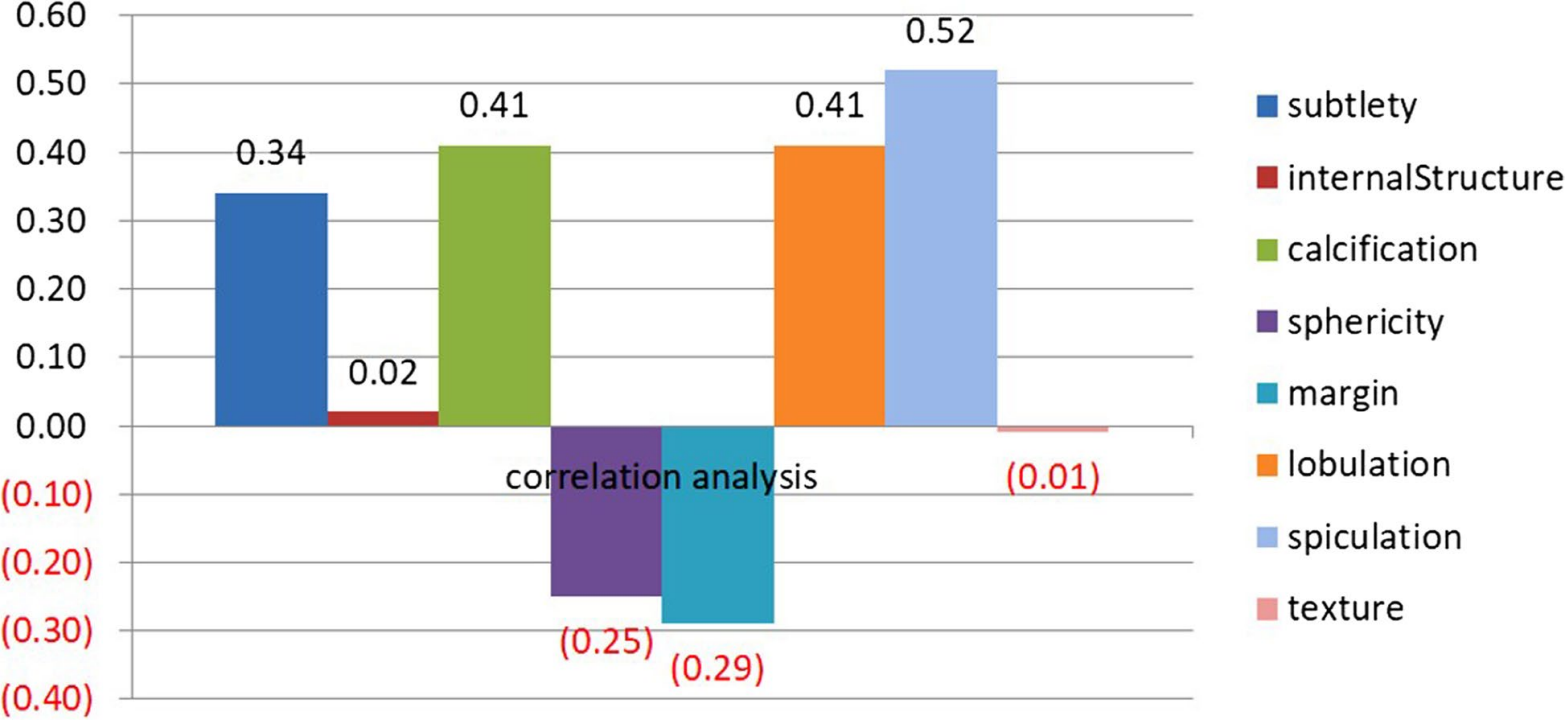
Correlation between eight characteristics and malignancy on 807 nodules

The correlation analysis result is depicted in Fig. 1. We can find that the ratings of SCs are positively correlated with malignancy except for sphericity and margin. Here, we calculate the absolute value of correlations to evaluate their correlations with malignancy. In this case, spiculation has the highest positive correlation with malignancy, followed by lobulation, calcification, subtlety, margin, sphericity, internal structure, and texture. Among the eight SCs, internal structure and texture have little influence on the rating of malignancy. Accordingly, we only focus on the grading of spiculation, lobulation, calcification, subtlety, margin, and sphericity.

### Neural network-based SC grading

In order to implement SC grading, we construct three neural network models in this section. The process includes two stages: preparation work and grading model construction.

#### Preparation work

##### Nodule SC rating normalization

Subtlety (C1), calcification (C3), sphericity (C4), margin (C5), lobulation (C6), and spiculation (C7) are important SCs related to malignancy (C9). Ratings of the six SCs were binarized by assigning a threshold at 3 to distinguish scores of 1–2 (Low level) from ratings of 3–6 (High level) shown in Table 4. A low level indicates a benign nodule labeled 0, and a high level denotes a malignant nodule marked 1. Ratings of calcification range from 1 to 6 and most of them are larger than 3. Accordingly, we binarized the ratings of calcification according to a threshold of 6, that is to say, the nodules with ratings of 6 were labeled 1 and others labeled 0.

**Table 4:** SC rating normalization

SCs	Low level	High level
Subtlety	Scale 1—2 Poor contrast between nodule and surroundings	Scale 3—5 High contrast between nodule and surroundings
Calcification	Scale 1—5 Present of calcification	Scale 6 Absent of calcification
Sphericity	Scale 1—2 Lesser roundness	Scale 3—5 Higher degree of roundness
Margin	Scale 1—2 Poorly defined margin	Scale 3—5 Sharper margin
Lobulation	Scale 1—2 None	Scale 3 – 5 Marked
Spiculation	Scale 1—2 None	Scale 3 – 5 Marked

##### Extraction of nodule image patches

We capture image patches of pulmonary nodules from CT images, and the principal steps are:

- Calculate the intersection region $R$, of nodule areas marked by up to 4 radiologists on CT image.
- Extract the minimum square bounding box $G$, around the region $R$.
- Enlarge $G$ with *n* = 5 pixels to retain more details of a nodule, especially its boundary information, and take $G$ as a grayscale nodule image.
- Calculate the corresponding binary nodule image $B$ with the same method from the region $R$.
- Normalize the sizes of $G$ and $B$ to 64 $\times$ 64.

**Fig. 2:**
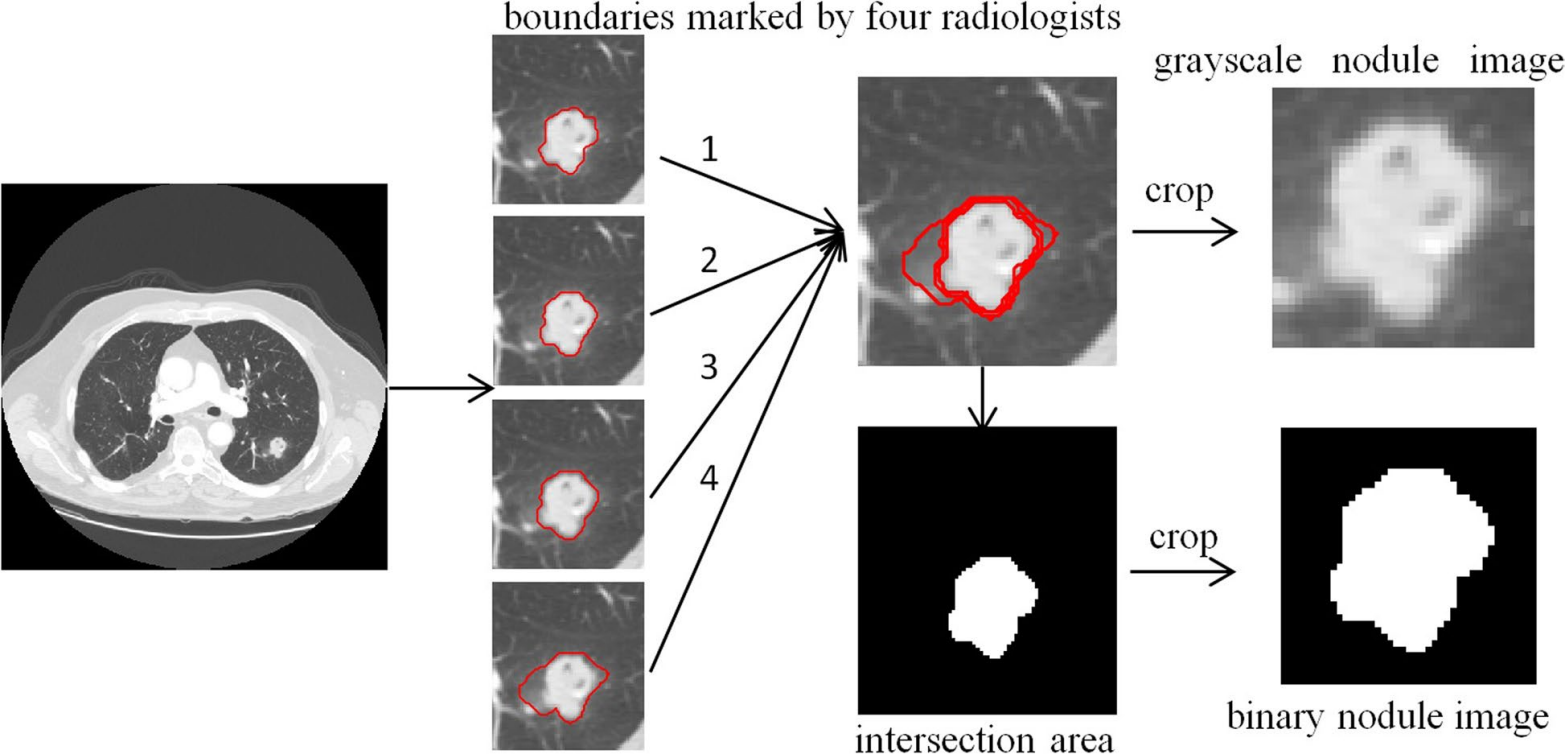
Extracting of nodule image patches

The process of nodule image patch extraction is described in Fig. 2.

#### Neural network model construction

We construct two classes of deep neural network-based classifiers for SC grading including deep belief network-based DBN model and convolutional neural network-based MBCNN model. The DBN model is further divided into DBN1 and DBN2 models according to different input types.

##### DBN model

**Fig. 3:**
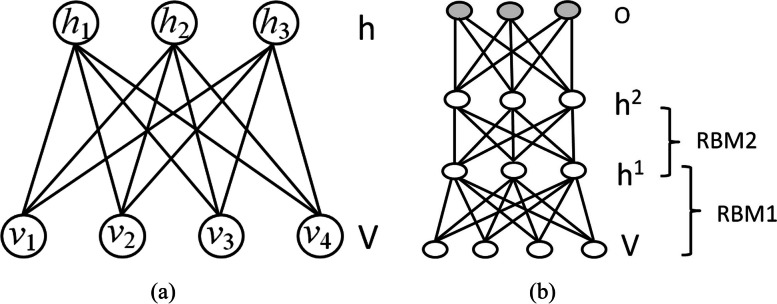
Architectures of RBM (**a**) and DBN (**b**)

DBN, a type of deep neural network, is widely used for feature generation and data classification. It is composed of several Restricted Boltzmann machines (RBMs) [29]. An RBM is a bipartite graph with an interlayer fully connected (see Fig. 3), and it usually contains two layers: a visible layer and a hidden layer. Its energy function is expressed as

2 $$E\left(v, h\right)={-a}^{T}v-{b}^{T}h-{v}^{T}Wh$$

where, $v$, $h$, $a$, $b$ and $w$ are respectively the visible layer, hidden layer, bias vector for the visible layer, bias vector for the hidden layer, and weight matrix defining the interaction between units of visible and hidden layers. RBM is trained to maximize the product of the probability of visible vectors based on the following energy function:

3 $$\arg\; \underset{W,a}{\max P\left(v\right)}=\frac1Z\sum\limits_h\exp\left(-E\left(v,h\right)\right)$$

where $Z$ is a partition function. A contrastive divergence algorithm combining Gibbs sampling and gradient descending is used to optimize the RBM.

**Fig. 4:**
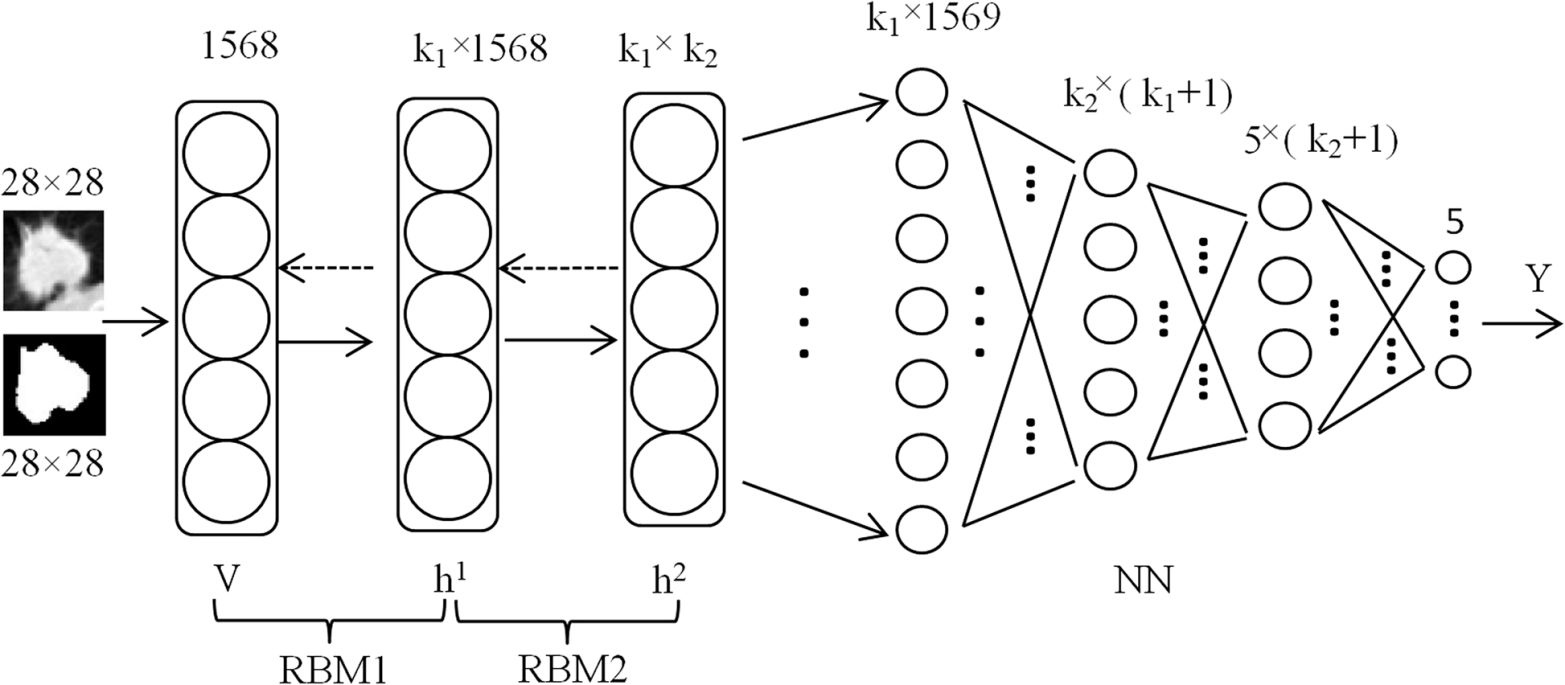
The architecture of the DBN model

The DBN model comprises two hidden layers with 100 nodes in each hidden layer as illustrated in Fig. 4.

Grayscale nodule images and corresponding binary nodule images are taken as the training and testing samples of DBN, and this DBN is called DBN1 in this study.

Image artificial features are the important basis for image analysis, and a total of 72 features are extracted from grayscale nodule image $G$ and binary nodule image $B$, as listed in Table 5, where, texture and intensity features are extracted from $G$ and geometric features from $B$. The grayscale nodule images and the corresponding features are taken as another sample set of our DBN model, and this DBN are called DBN2 in this study.

**Table 5:** Image features extracted from lung nodule images

Texture features [30–32]	Haralick features calculated from gray-level co-occurrence matrices, such as Autocorrelation, Contrast, Correlation, Cluster Prominence, Cluster Shade, Dissimilarity, Energy, Entropy, Homogeneity, Maximum probability, Sum of squares, Sum average, Sum variance, Sum entropy, Difference variance, Difference entropy and so on
	Image entropy
Geometric features	Centroid, MajorAxisLength, MinorAxisLength, Eccentricity, Orientation, ConvexArea, FilledArea, EulerNumber, EquivDiameter, EquivDiameter, Solidity, Extent, Perimeter, PerimeterOld
	Hu's invariant moments [33]
Intensity features	mean, variance, skewness

##### MBCNN model

**Fig. 5:**
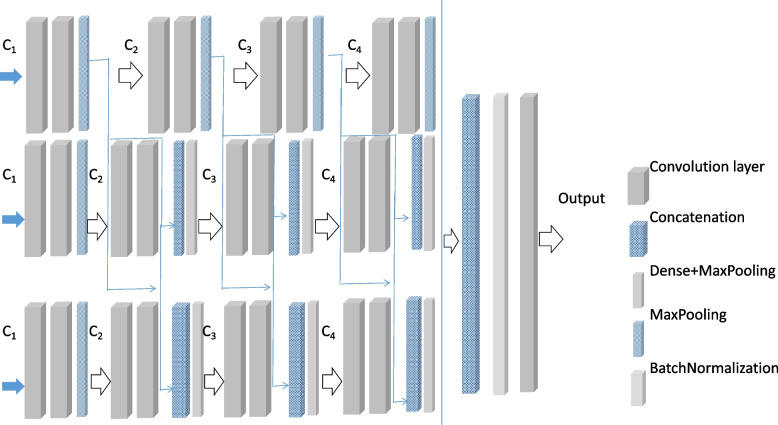
The architecture of the MBCNN model

CNN has the ability of representation learning and can carry out shift-invariant classification of input information according to its hierarchical structure. It has been achieved promising results in nodule classification taking raw image data as input without relying on a set of prior features. In this paper, we construct a multi-branch CNN classifier (MBCNN) shown in Fig. 5. The input of the model is three consecutive images of the same pulmonary nodules, and the output is the characteristic classification of pulmonary nodules. The objective is to extract comprehensive and accurate characteristic information of pulmonary nodules and improve the semantic classification accuracy of pulmonary nodules.

**Fig. 6:**
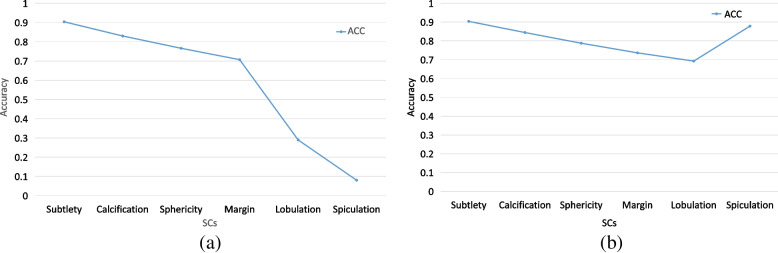
Average accuracy SC grading with DBN1 (**a**) and DBN2 (**b**) models

The input images are scaled to 64 × 64. Each branch is composed of multiple convolutional layers, pooling layers, connection layers, and dense layers. In the model, each convolution layer set (C_1_ ~ C_4_) covers two convolution layers with the same number of kernels. The convolution layer sets (C_1_ ~ C_4_) consisted of 16, 32, 64, and 128 kernels of size 3 × 3, respectively. In the first branch, each convolutional layer set is followed by 2 × 2 max-pooling layers with a stride of 2. In the second branch, the convolution layer sets from the first branch are concatenated to the corresponding convolution layer sets as shown in Fig. 6. Similarly, in the third branch, the convolution layer sets from the first and second branches are concatenated to the corresponding convolution layer sets. Multiple inputs can enrich features. More, by adding skip connections, more information has the opportunity to be retained, increasing the generalization ability of the model and reducing the risk of overfitting.

After the output vectors of each branch are obtained, they are merged through cascade operation and then batch-normalized. The result is the output of a full connection layer with 64 kernels of size 3 × 3, which results in SC classification of pulmonary nodules after dense layers.

## Results

To evaluate the proposed method, we randomly select 1724 pulmonary nodules from the LDC-IDRI, and grade six SC ratings with our neural network-based SC grading models. We take Accuracy (ACC), Recall (REC), Area under curve (AUC), and Dice similarity coefficient (DSC) as the metrics for evaluating the accuracy of SC grading [34, 35].

ACC represents the ratio of the number of correctly classified samples to the total number of samples participating in the classification. REC is defined as the ratio of the number of samples correctly classified as true to the number of original true samples. ACC and REC are defined as

4 $$\mathrm{ACC}=\frac{TP+TN}{TP+TN+FP+FN}$$

5 $$\mathrm{REC}=\frac{TP}{TP+FN}$$

where, TP (True Positive), FP (False Positive), TN(True Negative), and FN(False negative) are the number of samples correctly classified as true, incorrectly classified as true, correctly classified as false, and incorrectly classified as false, respectively.

AUC index is used to assess the classifier's ability to distinguish between positive and negative samples:

6 $$AUC=\frac{{\Sigma }_{i \epsilon positive}{rank}_{i}-M\times \left(M+1\right)/2}{M\times N}$$

where $M$, $N$ are the number of positive and negative samples, respectively. ${rank}_{i}$ is the order of positive sample probabilities

DSC is a set similarity measure function that is usually used to calculate the similarity of two samples, where DSC is defined as

7 $$DSC\left(A, B\right)=2\times \left|A\cap B\right|/\left(\left|A\right|+\left|B\right|\right)$$

where $|A|$ and $|B|$ are the number of samples $A$ and $B$, respectively. $|A \cap B|$ is samples in the intersection of $A$ and $B$.

### DBN model evaluation

The DBN1 model takes grayscale and binary nodule images as the input, and the DBN2 model uses grayscale nodule images and corresponding features as the input. We list their training accuracy of fivefold cross-validation results in terms of ACC in Tables 6 and 7, and average accuracy in Fig. 6(a) and Fig. 6(b), respectively.

**Table 6:** The evaluation result of SC grading with the DBN1 model

SCs	1	2	3	4	5
Subtlety	0.888	0.928	0.862	0.880	0.962
Calcification	0.805	0.865	0.797	0.822	0.862
Sphericity	0.772	0.802	0.725	0.725	0.807
Margin	0.718	0.747	0.672	0.670	0.728
Lobulation	0.780	0.128	0.160	0.163	0.218
Spiculation	0.140	0.078	0.065	0.043	0.075

**Table 7:** The evaluation result of SC grading with the DBN2 model

SCs	1	2	3	4	5
Subtlety	0.888	0.928	0.862	0.880	0.962
Calcification	0.805	0.937	0.797	0.822	0.862
Sphericity	0.772	0.910	0.725	0.725	0.807
Margin	0.718	0.895	0.672	0.670	0.728
Lobulation	0.220	0.785	0.840	0.837	0.782
Spiculation	0.860	0.717	0.935	0.957	0.925

Table 6 lists the training grading accuracy of six SCs with the DBN1 model. From Table 6, we can find that the DBN1 method can well grade subtlety with an accuracy of 0.904, while it can’t classify lobulation and spiculation successfully. The average ACC (Ave) is 0.596 on the grading of six SCs as shown in Fig. 6(a). Table 7 shows the grading results of SCs with the DBN2 model. It can be seen that the grading accuracy of subtlety and spiculation are more than 0.9, and the DBN2 model has a high Ave (0.807) on the grading of six SCs. In addition, it can be observed from Fig. 6(b) that DBN2 achieves higher accuracy on gradings of calcification, sphericity, margin, lobulation, and spiculation than DBN1. So the features extracted from pulmonary nodule images are helpful for SC grading.

### MBCNN model evaluation

MBCNN model can automatically extract deep features from pulmonary nodule images and classify SCs with them. The evaluation result of the grading of six SCs is shown in Table 8. It can be observed that the grading accuracy of subtlety, calcification, sphericity and spiculation are all higher than 0.9, and the Ave achieves 89.37%.

**Table 8:** The evaluation result of SC grading with the MBCNN model

	**Subtlety**	**Calcification**	**Sphericity**	**Margin**	**Lobulation**	**Spiculation**
ACC	0.9800	0.9925	0.9825	0.6625	0.7649	0.9800
SEN	0.9783	9903	0.9807	0.6538	0.7548	0.9783
DSC	0.9821	9872	0.9807	0.6554	0.7465	0.9783
AUC	0.9968	9947	0.9825	0.7379	0.8244	0.9824

**Fig. 7:**
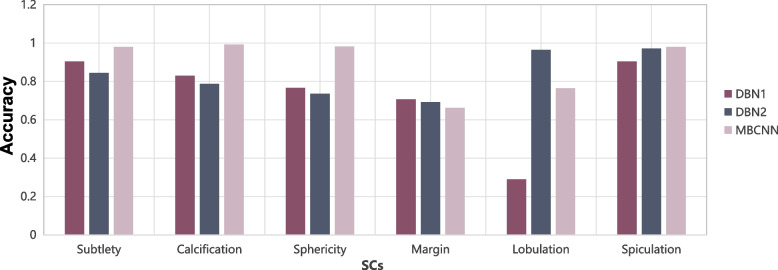
Comparison of three models on SC grating

In order to visualize the rating results of the three methods, we depict the rating evaluation results of six SCs in terms of ACC, and see Fig. 7 for a reference. We can find that the average accuracy of the MBCNN method is higher than those of the other two methods. In DBN-based methods, the average accuracy of DBN2 is higher than that of DBN1. That is to say, features extracted from nodule images are more effective than those automatically learned by the DBN1 model. MBCNN model is superior to other models benefiting from the design of multi-branch structures, deep learning, skip connections.

## Discussion

**Fig. 8:**
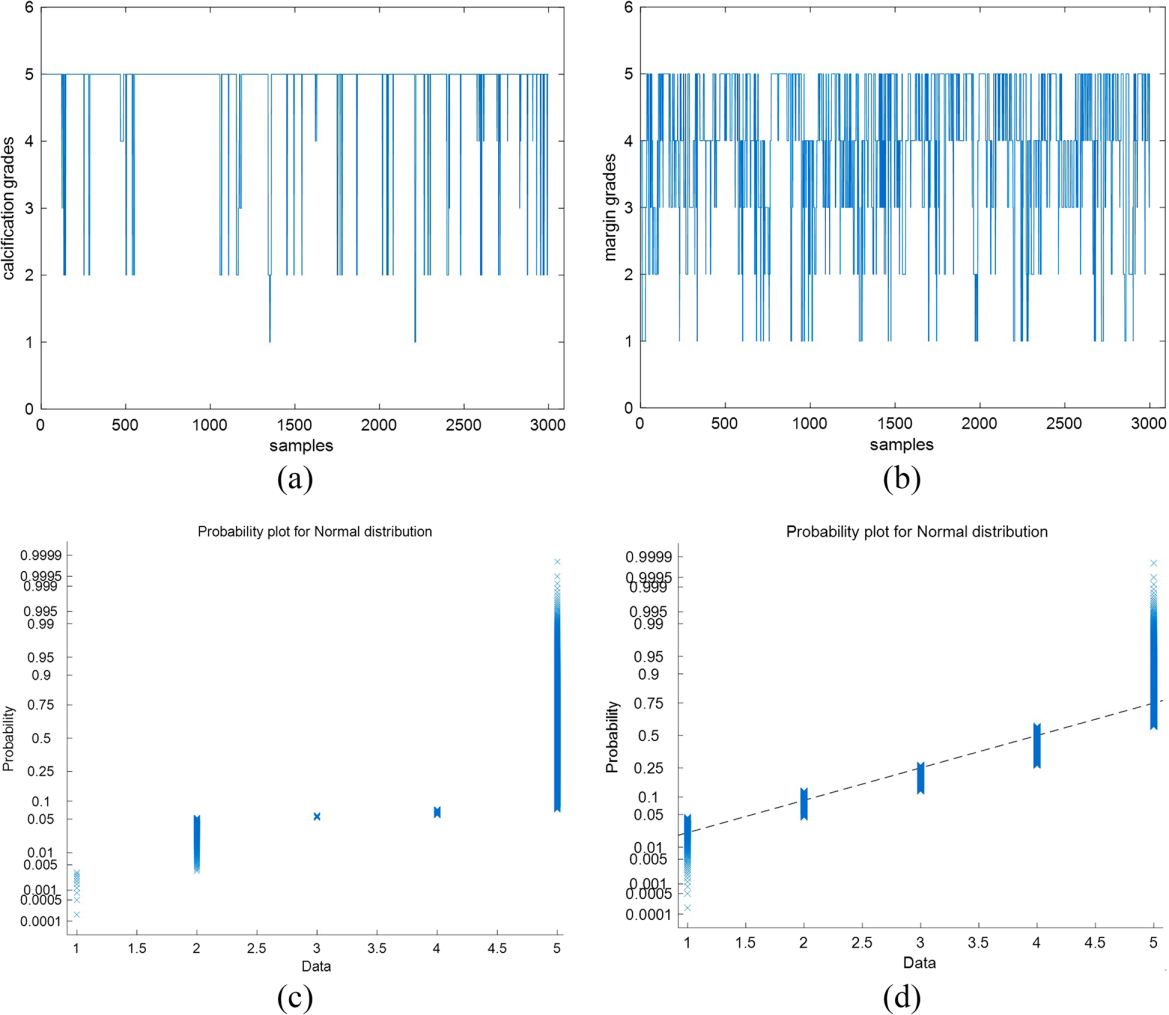
Characteristic rating analysis of calcification (**a**) and margin (**b**). Images on the top row are rating distributions and their corresponding normal probability plots on the bottom row

Firstly, it can be found from the experiments that the classification accuracies of the SC grading vary greatly. For example, the accuracy of calcification grading is higher than that of margin. We check their ratings marked by radiologists and plot the rating distributions. The corresponding normal probability plots are depicted in Fig. 8. From Fig. 8, we can find that the rating distribution of calcification is more regular than that of margin. Ratings of calcification are mainly 1 and 6 (see Fig. 8(a)), and ratings of margin are located in a range from 1 to 5 (see Fig. 8(b)). Therefore, the original rating distribution of SCs in the sample set is an important factor influencing grading SCs.

**Fig. 9:**
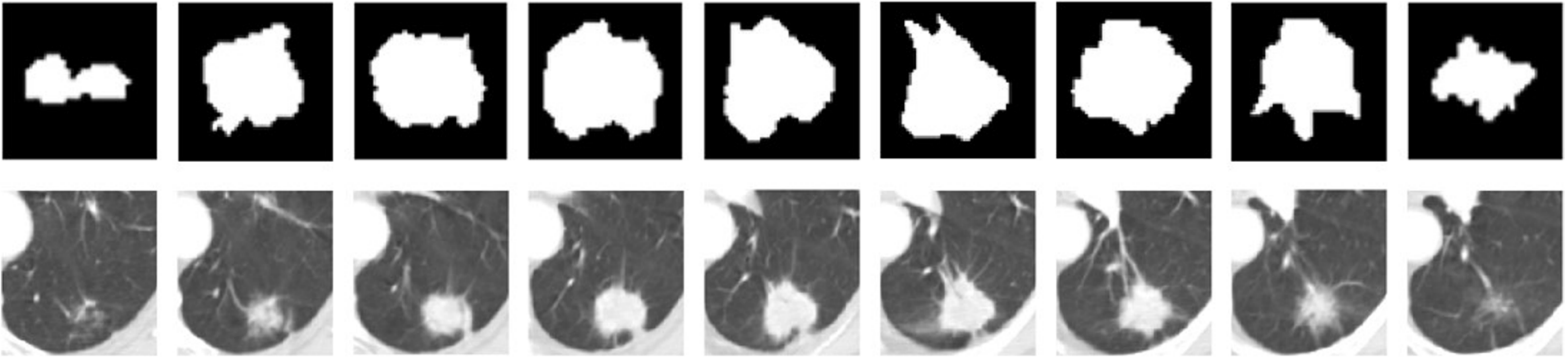
Images (top row) and the corresponding binary shapes (bottom row) of a nodule selected from a series of CT slices

Secondly, the MBCNN model can generate higher accuracy than the DBN-based models. We randomly select a nodule from LIDC-IDRI and analyze its SC ratings provided by four radiologists, as displayed in Table 9. A CT scanner samples the nodule and its imaging is distributed in a set of slices. It can be seen from Fig. 9 that the shapes of a nodule are various in different slices. However, they all share the same set of SC ratings annotated by a radiologist because they belong to the same nodule. Consequently, SC grading using one two-dimensional image of pulmonary nodules can result in less-than-ideal results. The multi-image of a nodule will be more beneficial for grading SCs with machine learning techniques. At the same time, there is disagreement among the radiologists on the grading of SCs and their drawn outlines of the extent of nodules, which hampers the establishment of standard training and testing datasets. There usually be disagreement among the radiologists on the grading of SCs and their drawn outlines of the extent of nodules. In addition, the SC classification relies only on the radiologists’ naked eye observation without further pathological examination. All of these factors influence the establishment of more standard training and testing datasets to a certain degree.

**Table 9:** SC grading by four radiologists on a pulmonary nodule

SCs	C1	C2	C3	C4	C5	C6	C7	C8	C9
No.1	5	1	6	3	3	3	4	5	5
No.2	5	1	6	4	4	5	5	5	5
No.3	5	1	6	3	2	3	3	5	5
No.4	5	1	6	5	4	1	5	4	4

## Conclusions

Automatic grading of pulmonary nodule SCs plays a vital role in CADx, and it is helpful for radiologists to determine the probabilities of the likelihood of malignancy of a pulmonary nodule. In this paper, we analyze the SCs of nodules and find that spiculation has the highest correlation with the malignancy of pulmonary nodules among eight SCs, followed by lobulation, calcification, subtlety, margin, sphericity, internal-Structure, and texture. DBN model and MBCNN model are used to grade SCs, and some meaningful results are obtained on the SC grading. We further discuss the grading results and give suggestions on the SC grading. In the future, we try to improve the performance of our algorithm by extracting three-dimensional features of pulmonary nodules on one hand, optimizing the model architecture with a more advanced baseline, such as VGG16, combining attention mechanism, skip connections, etc., to further improve the classification ability.

## Data Availability

The datasets analyzed during this current study are available in the public LIDC-IDRI database with a website of https://wiki.cancerimagingarchive.net/display/Public/LIDC-IDRI
